# Two Cases of Autochthonous West Nile Virus Encephalitis, Paris, France, 2025

**DOI:** 10.3201/eid3111.251220

**Published:** 2025-11

**Authors:** Nolan Hassold-Rugolino, Pierre Jaquet, Daniel Da Silva, Evangelina Papa, Julie Calmettes, Carole Henry, Hilaire Flamant, Etienne Brière, Guillaume André Durand, Gilda Grard, Ines Jabnoune, Nelly Fournet, Kamel Harchaou, Alexandre Bleibtreu, Michelle Saliba, Aude Gibelin, Marion Parisey, Yacine Tandjaoui-Lambiotte

**Affiliations:** Centre Hospitalier de Saint-Denis Hôpital Delafontaine, Saint-Denis, France (N. Hassold-Rugolino, P. Jaquet, D. Da Silva, E. Papa, J. Calmettes, C. Henry, H. Flamant, E. Brière, I. Janoune, M. Saliba, A. Gibelin, M. Parisey, Y. Tandjaoui-Labiotte); French Armed Forces Biomedical Research Institute, Valérie-André, Brétigny-sur-Orge, France (G.A. Durand, G. Grard); Aix-Marseille University, Marseille, France (G.A. Durand, G. Grard); Santé Publique France, Saint Maurice, France (N. Fournet); CHU Pitié-Salpêtrière, Paris, France (A. Bleibtreu)

**Keywords:** West Nile virus, viruses, zoonoses, meningitis/encephalitis, vector-borne infections, mosquitoes, Paris, France

## Abstract

We report 2 cases of febrile lymphocytic meningitis with encephalitis in patients in France. One patient had not traveled outside Paris; the other had traveled to eastern France. Laboratory findings revealed acute West Nile virus infection. The cases occurred days apart, raising concern the virus has spread further in France.

West Nile virus (WNV) is an orthoflavivirus transmitted primarily by *Culex* spp. mosquitoes, which are endemic in France (*1*). Recent data also suggest possible transmission by *Aedes albopictus* mosquitoes, which are now endemic in the greater Paris area, representing a potential new WNV vector in France (*2*). 

Discovered in 1937 in Uganda, WNV has spread worldwide; in 1999, it was detected in the United States, where it is now endemic in nearly all states (*3*). A major epidemic occurred in Europe in 2018, affecting the Mediterranean coast, and autochthonous WNV cases have been reported in France in Camargue, in the Var and Aquitaine region (*4*–*6*). We report 2 autochthonous WNV cases in Paris occurring just days apart. 

The first patient was a 64-year-old previously healthy man who arrived at the emergency department (ED) on July 25, 2025, with a 1-week history of asthenia, febrile headache, and altered mental status. His last travel outside France was to Portugal in 2016; he had stayed in the Jura department in eastern France 3 weeks earlier. Initial lumbar puncture exhibited neutrophil-predominant meningitis (leukocytes, 430 cells/L [reference <5 cells/L] with 76% neutrophils) and elevated cerebrospinal fluid (CSF) protein (1.19 g/L [reference <0.40 g/L]) without hypoglycorrhachia (Table). A multiplex neuromeningeal PCR panel using QIAstat-Dx (QIAGEN, https://www.qiagen.com) was negative. Probabilistic treatment with intravenous ceftriaxone, amoxicillin, acyclovir, and dexamethasone was initiated in the ED before the patient was admitted to the intensive care unit (ICU). A second lumbar puncture performed on hospitalization day 4 revealed lymphocytic meningitis (leukocytes, 48 cells/L with 88% lymphocytes). Brain magnetic resonance imaging results were unremarkable. Blood and CSF samples were both WNV positive by PCR and WNV-specific IgM.

**Table:** Clinical characteristics for 2 cases of autochthonous West Nile virus, Paris, France*

Characteristics	Patient 1	Patient 2
Age, y/sex	64/M	25/F
History of travel	Portugal (2016), Jura (in eastern France) 3 weeks earlier	None
Clinical manifestations	Fever, headache, obnubilation, neck stiffness	Fever, headache, peripheral facial paralysis, nystagmus, decreased reflexes in lower limbs, urinary retention
First lumbar puncture		
Protein level, g/L (reference <0.40)	430	1,200
Leukocyte count, cells/L (reference <0.05)	1.19; 76% neutrophils	1.95; 90% lymphocytes
Second lumbar puncture, time after admission	4 d	72 h
Protein level, g/L (reference <0.40)	48	260
Leukocyte count, cells/L (reference <5)	1.26; 88% lymphocytes	1.4; 75% lymphocytes
Brain MRI	Unremarkable	Unremarkable
PCR WNV	Positive in blood and CSF	Positive in blood and CSF
IgM WNV	Positive in blood and CSF	Positive in blood and CSF

*CSF, cerebrospinal fluid; MRI, magnetic resonance imaging; WNV, West Nile virus.

The second patient was a 25-year-old woman without any travel outside the Paris area during the past 4 years. She arrived at the ED on August 2, 2025, with fever, neck stiffness, binocular diplopia, and vertigo without altered mental status, and had a 3-day history of influenza-like illness. Lumbar puncture showed lymphocytic meningitis (leukocytes, 1,200 cells/L with 90% lymphocytes) with elevated CSF protein (1.95 g/L) without hypoglycorrhachia (Table); she also had biochemical evidence of hepatic cytolysis. Probabilistic treatment with intravenous cefotaxime (4 g/6 h), amoxicillin (2.5 g/6 h), 1 intravenous injection of gentamicin (5 mg/kg), acyclovir (600 mg/8 h), and dexamethasone (10 mg/6 h) was initiated in ED before ICU admission. Multiplex neuromeningeal PCR panel (QIAstat-Dx) was negative on first CSF sample. Brain magnetic resonance imaging results were unremarkable. A second lumbar puncture at 72 hours still showed lymphocytic meningitis with elevated CSF protein and normal glucose. Clinical examination evolved to include static and kinetic cerebellar syndrome with multidirectional nystagmus, bilateral peripheral facial nerve paralysis, decreased deep tendon reflexes in the lower limbs, and acute urinary retention. Both blood and CSF were WNV positive by PCR and IgM testing.

Neither patient had underlying immunodepression, and both had low inflammatory syndrome (C reactive protein <30 mg/L) with negative procalcitonin. WNV RNA was detected by using a specific real-time duo reverse transcription PCR, and IgM was detected by using the Anti-West Nile Virus ELISA on the Analyzer I-2P platform (both EUROIMMUN, https://www.euroimmun.com) at the national reference center for France. Others flaviviruses, notably Usutu virus, were ruled out.

The cases described raise concern in France, particularly because, although the patients were immunocompetent and had favorable outcomes, the literature suggests a high (10%) mortality rate for neuroinvasive forms of WNV and worse outcomes for immunocompromised patients (*3*,*7*). Of note, clinical manifestations varied between the 2 cases: 1 patient had acute encephalitis with altered mental status, typical of WNV infection; the other had rhombencephalitis without altered consciousness but bilateral peripheral facial paralysis and cerebellum involvement, uncommon but previously described manifestations of the disease (*8*).

These cases should not be overlooked because no human vaccine or effective therapy exists for WNV infection. Preliminary results from a randomized trial in Israel investigating convalescent plasma to treat WNV found a 10% reduction in mortality rates for each 10-fold increase in neutralizing antibody titer (*9*).

A hypothesis to explain viral dissemination, notably in the Nouvelle Aquitaine region of France, involves climate change–related alterations to avian migratory patterns. Environmental and spatioenvironmental risk models confirm that the structure of migratory bird flyways contributes to the geographic dynamics of human WNV outbreaks in Europe, particularly in river basins (the 2 patients lived near the Seine) and wetland areas, further underscoring the One Health concept in the human dissemination of pathogens (*10*). Phylogenetic analyses comparing the strains from these 2 cases with other strains circulating in France to determine WNV lineage are ongoing.

In conclusion, our findings highlight spread of WNV into northern France during the mosquito season. This new area of WNV circulation is a concern for physicians managing infected patients but also for blood, cell, or organ donations for which qualified donors are needed. In addition, the 2 cases likely indicate northward spread of the virus beyond its previously known range. Clinicians should be aware of WNV in the greater Paris region and test any patient with unexplained lymphocytic meningitis.
